# 手性毛细管气相色谱法拆分4-氯甲基-2,2-二甲基-1,3-二氧戊环对映异构体

**DOI:** 10.3724/SP.J.1123.2023.06010

**Published:** 2023-12-08

**Authors:** Zhenyong ZHANG

**Affiliations:** 岛津(上海)实验器材有限公司,上海200233; Shimadzu (Shanghai) Global Laboratory Consumables Co., Ltd., Shanghai 200233, China

**Keywords:** 气相色谱法, 手性拆分, 4-氯甲基-2,2-二甲基-1,3-二氧戊环, 对映异构体, gas chromatography (GC), chiral separation, 4-chloromethyl-2,2-dimethyl-1,3-dioxolane, enantiomers

## Abstract

手性化合物具有独特的生物活性,其在药物治疗中发挥着关键作用。为准确测定对映异构体中不同构型的纯度,建立手性化合物对映体分离方法十分关键。4-氯甲基-2,2-二甲基-1,3-二氧戊环是一种重要的手性医药中间体,本文建立了一种手性毛细管气相色谱拆分测定4-氯甲基-2,2-二甲基-1,3-二氧戊环对映异构体的方法。采用固定相为环糊精衍生物的色谱柱Rt-bDEXse进行分离,并以氢火焰离子化检测器进行检测。经过优化,得到最优色谱条件:线速度为70 cm/s;升温程序为初始柱温70 ℃保持1 min,以2.0 ℃/min的速率升温到150 ℃;溶样溶剂为甲醇。实验结果表明,在该条件下,(*R*)-4-氯甲基-2,2-二甲基-1,3-二氧戊环和(*S*)-4-氯甲基-2,2-二甲基-1,3-二氧戊环可在10 min内快速分离,两者的分离度远大于1.5,在0.5~50.0 mg/L的范围内线性关系良好,线性相关系数均大于0.998, (*R*)-和(*S*)-4-氯甲基-2,2-二甲基-1,3-二氧戊环的检出限分别为0.07和0.08 mg/L,定量限分别为0.22 mg/L和0.25 mg/L。以甲醇作为空白样品,进行0.5、2.0、10.0 mg/L 3个不同水平的加标回收试验,(*R*)-和(*S*)-4-氯甲基-2,2-二甲基-1,3-二氧戊环的回收率分别为94.0%~99.1%和96.0%~98.8%,相对标准偏差(RSD)分别为1.26%~4.87%和1.51%~4.46%。该方法可为手性医药中间体4-氯甲基-2,2-二甲基-1,3-二氧戊环对映异构体的拆分提供参考。

## Abstract (en)

Chiral compounds play an important role in the pharmaceutical industry owing to their unique biological activities. The enantiomers must be separated because they can exhibit different pharmacological activities. Thus, the development of chiral separation methods is essential to determine the purity of enantiomers. 4-Chloromethyl-2,2-dimethyl-1,3-dioxolane is an important chiral pharmaceutical intermediate. In this context, a method based on chiral capillary gas chromatography was established for the separation and determination of the enantiomers of 4-chloromethyl-2,2-dimethyl-1,3-dioxolane. The separation of (*R*)- and (*S*)-4-chloromethyl-2,2-dimethyl-1,3-dioxolane was initially investigated using two conventional stationary-phase capillary columns: SH-I-5Sil MS and SH-WAX. The stationary phase of SH-I-5Sil MS consisted of 5% phenyl and 95% polymethylsiloxane, whereas the stationary phase of SH-WAX consisted of 100% crosslinked polyethylene glycol. Neither of the columns exhibited chiral selectivity, so they both were unable to separate the enantiomers of 4-chloromethyl-2,2-dimethyl-1,3-dioxolane. Subsequently, the separation of (*R*)- and (*S*)-4-chloromethyl-2,2-dimethyl-1,3-dioxolane was investigated using four chiral columns: Rt-bDEXm, Rt-bDEXsm, Rt-bDEXse, and InertCap CHIRAMIX. Among the chiral columns, Rt-bDEXse, which used a stationary phase composed of 2,3-di-*O*-ethyl-6-*O*-*tert*-butyl dimethylsilyl *β*-cyclodextrin added to 14% cyanopropyl phenyl and 86% dimethyl polysiloxane, achieved the best separation of (*R*)- and (*S*)-4-chloromethyl-2,2-dimethyl-1,3-dioxolane. Thus, this column was selected as the analytical column for further method optimization. Detection was performed using a hydrogen flame ionization detector. The effects of various gas chromatographic parameters, such as linear velocity, initial column temperature, column heating rate, and solvent type, on the separation of (*R*)- and (*S*)-4-chloromethyl-2,2-dimethyl-1,3-dioxolane were investigated. The optimal chromatographic conditions included a linear velocity of 70 cm/s, an initial column temperature of 70 ℃, and a column heating rate of 2.0 ℃/min. The final column oven temperature was 150 ℃. Methanol, ethanol, ethyl acetate, *n*-hexane, dichloromethane, and dimethyl sulfoxide were selected as solvents. The results showed that dimethyl sulfoxide interfered with the peaks of the target compounds, whereas the other solvents had no significant effect on the peak shape and separation of (*R*)- and (*S*)-4-chloromethyl-2,2-dimethyl-1,3-dioxolane. Methanol was finally selected as the solvent in this study. Further experiments revealed that (*R*)- and (*S*)-4-chloromethyl-2,2-dimethyl-1,3-dioxolane could be rapidly separated within 10 min, with a resolution greater than 1.5. A good linear relationship was observed in the range of 0.5-50.0 mg/L, with a linear correlation coefficient greater than 0.998. The limits of detection for (*R*)- and (*S*)-4-chloromethyl-2,2-dimethyl-1,3-dioxolane were 0.07 and 0.08 mg/L, respectively, and the corresponding limits of quantification were 0.22 and 0.25 mg/L, respectively. Spiked recovery tests were performed at three spiked levels of 0.5, 2.0, and 10.0 mg/L using methanol as the blank to determine the accuracy of the proposed method. The recoveries for (*R*)- and (*S*)-4-chloromethyl-2,2-dimethyl-1,3-dioxolane were 94.0%-99.1% and 96.0%-98.8%, respectively, with relative standard deviations (RSDs) of 1.26%-4.87% and 1.51%-4.46%, respectively. The established method is efficient and reliable; thus, it can serve as a reference for the separation of the enantiomers of 4-chloromethyl-2,2-dimethyl-1,3-dioxolane. It can also potentially be applied to evaluate the enantiomeric purity of other chiral compounds in the pharmaceutical industry and produce chiral drugs and other related compounds.

(*R*)-4-氯甲基-2,2-二甲基-1,3-二氧戊环和(*S*)-4-氯甲基-2,2-二甲基-1,3-二氧戊环可作为有机合成中间体和手性有机砌块,用于手性化工产品的合成过程^[1⇓⇓-4]^,其结构式见图1。

**图1:**
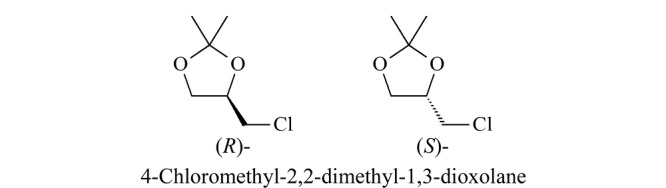
(*R*)-和(*S*)-4-氯甲基-2,2-二甲基-1,3-二氧戊环的结构式

Babu等^[3]^使用(*S*)-4-氯甲基-2,2-二甲基-1,3-二氧戊环作为手性合成砌块,成功合成了抗菌药利奈唑胺。Bhavani等^[4]^使用(*R*)-环氧氯丙烷合成中间体(*R*)-4-氯甲基-2,2-二甲基-1,3-二氧戊环,进一步合成了有机砌块(*S*)-3-氨基-1,2-丙二醇。为了更好地研究合成工艺并进一步测定合成物手性组分含量,拆分4-氯甲基-2,2-二甲基-1,3-二氧戊环对映异构体变得尤为必要。

针对对映体的拆分,主要方法有超高效合相色谱法^[5⇓⇓-8]^、高效液相色谱法^[9⇓⇓⇓-13]^和气相色谱法^[14⇓-16]^。目前尚未见关于4-氯甲基-2,2-二甲基-1,3-二氧戊环对映异构体拆分的相关报道。本文采用手性气相色谱法建立了4-氯甲基-2,2-二甲基-1,3-二氧戊环对映异构体拆分方法,并对色谱柱类型、载气线速度、初始柱温、升温速率、溶样溶剂种类等色谱条件进行了详细优化。建立的方法简单快速,灵敏度高,可为手性中间体4-氯甲基-2,2-二甲基-1,3-二氧戊环对映异构体的拆分提供参考。

## 1 实验部分

### 1.1 仪器、试剂与材料

GC-2030气相色谱仪、色谱工作站、AT-R电子天平(日本岛津公司);PR-FP-0120α-MT1纯水机(日本奥加诺公司)。

甲醇、乙醇、乙酸乙酯、正己烷、二氯甲烷、二甲基亚砜(色谱纯), (*R*)-4-氯甲基-2,2-二甲基-1,3-二氧戊环(纯度≥98.0%)、(*S*)-4-氯甲基-2,2-二甲基-1,3-二氧戊环(纯度≥97.0%),购买于上海安谱实验科技股份有限公司。外消旋体标准品,自行配制,由上述(*R*)-4-氯甲基-2,2-二甲基-1,3-二氧戊环和(*S*)-4-氯甲基-2,2-二甲基-1,3-二氧戊环按质量比1∶1混合制成。

### 1.2 标准溶液的配制

准确称量20.0 mg外消旋体标准品,用甲醇溶解定容至10 mL,制成质量浓度为2.0 mg/mL的外消旋体标准储备液。取适量外消旋体储备液,用甲醇稀释至200 mg/L,作为外消旋体标准使用液。

分别准确称量10.0 mg(*R*)-4-氯甲基-2,2-二甲基-1,3-二氧戊环和(*S*)-4-氯甲基-2,2-二甲基-1,3-二氧戊环标准品,用甲醇稀释定容至100 mL,制成质量浓度为100 mg/L的单标标准储备液。分别移取两者适量的单标标准储备液,用甲醇逐级稀释配制质量浓度为50.0、20.0、10.0、5.0、2.0、1.0、0.5 mg/L的系列混合标准溶液。

### 1.3 气相色谱条件

色谱柱为Rt-bDEXse(30 m×0.25 mm×0.25 μm);进样口温度为200 ℃;分流进样,分流比为9∶1;恒线速度模式,线速度为70 cm/s;氢火焰检测器温度为200 ℃;氢气流量为30 mL/min;空气流量为300 mL/min;进样量为1 μL;载气为氦气。升温程序如下:初始温度70 ℃保持1 min,以2.0 ℃/min的速率升温至150 ℃。

## 2 结果与讨论

### 2.1 色谱柱的选择

取4-氯甲基-2,2-二甲基-1,3-二氧戊环外消旋体标准使用液进行上机测试,采用SH-I-5Sil MS(固定相为5%苯基-95%聚甲基硅氧烷,30 m×0.25 mm×0.25 μm)和SH-WAX(固定相为100%键合交联聚乙二醇,30 m×0.25 mm×0.25 μm)色谱柱作为分析柱时,4-氯甲基-2,2-二甲基-1,3-二氧戊环对映异构体完全无分离趋势。表明常规聚硅氧烷类固定相或聚乙二醇类固定相对该手性异构体几乎没有特征选择性。

采用Rt-bDEXm(固定相为全甲基化*β*-环糊精掺杂的14%氰基丙基苯基-86%二甲基聚硅氧烷,30 m×0.25 mm×0.25 μm)、Rt-bDEXsm(固定相为2,3-二-*O*-甲基-6-*O*-叔丁基二甲基硅基*β*-环糊精掺杂的14%氰基丙基苯基-86%二甲基聚硅氧烷,30 m×0.25 mm×0.25 μm)、Rt-bDEXse(固定相为2,3-二-*O*-乙基-6-*O*-叔丁基二甲基硅基*β*-环糊精掺杂的14%氰基丙基苯基-86%二甲基聚硅氧烷,30 m×0.25 mm×0.25 μm)和InertCap CHIRAMIX(固定相为*β*-环糊精和*γ*-环糊精混合物,30 m×0.25 mm×0.25 μm)色谱柱作为分析柱进行测试,使用相同的初始色谱条件,即线速度为50 cm/s,升温程序为40 ℃保持1 min,以2.0 ℃/min的速率升温至150 ℃。4-氯甲基-2,2-二甲基-1,3-二氧戊环外消旋体标准使用液在4款色谱柱上的保留和分离情况见图2。根据实验结果,Rt-bDEXse色谱柱对(*R*)-和(*S*)-4-氯甲基-2,2-二甲基-1,3-二氧戊环的分离效果最佳,且两者的色谱响应最高。因此后续将采用该色谱柱进行方法优化。

**图2:**
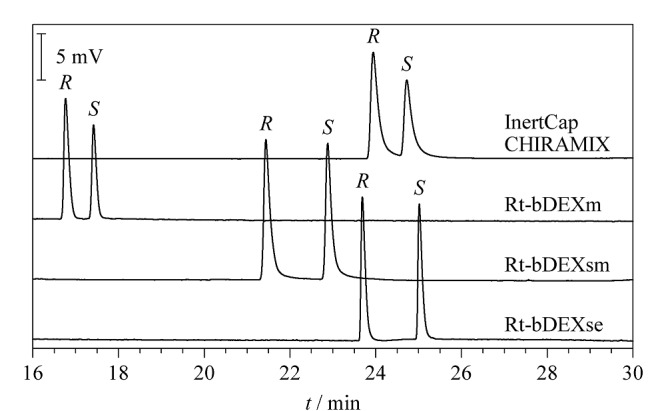
(*R*)-和(*S*)-4-氯甲基-2,2-二甲基-1,3-二氧戊环在不同色谱柱上的色谱图

### 2.2 线速度对分离度的影响

本文考察了线速度为30、40、50、60、70、80、90 cm/s时,(*R*)-和(*S*)-4-氯甲基-2,2-二甲基-1,3-二氧戊环的分离度,见图3。结果表明,载气的线速度对分离度有显著影响。在载气线速度为70 cm/s以下时,随着线速度的增加,(*R*)-和(*S*)-4-氯甲基-2,2-二甲基-1,3-二氧戊环的分离度有所增加,在载气线速度为70 cm/s时分离度最大。且随着线速度的增加,化合物出峰时间会加快,同时峰形也会更加尖锐。但线速度在80 cm/s及以上时,两者间分离度迅速下降。因此实验最终选择载气线速度为70 cm/s。

**图3:**
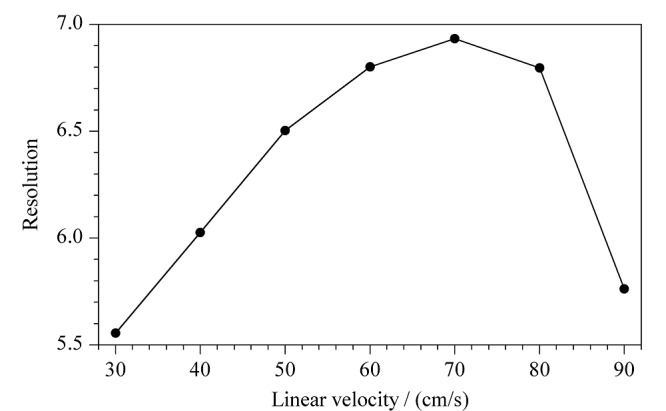
(*R*)-和(*S*)-4-氯甲基-2,2-二甲基-1,3-二氧戊环 在不同线速度下的分离度

### 2.3 初始柱温对分离度的影响

本文考察了初始柱温为30、40、50、60、70、80、90及100 ℃时(*R*)-和(*S*)-4-氯甲基-2,2-二甲基-1,3-二氧戊环的分离度,见图4。结果表明,在初始柱温为70 ℃以上时,(*R*)-和(*S*)-4-氯甲基-2,2-二甲基-1,3-二氧戊环的分离度显著下降,且两者的保留时间会显著缩短。在初始柱温为70 ℃以下时,继续降低初始柱温,分离度不再有更明显的提升,但明显延长了分析时间。为了平衡分离度和分析时长,实验最终选择初始柱温为70 ℃。

**图4:**
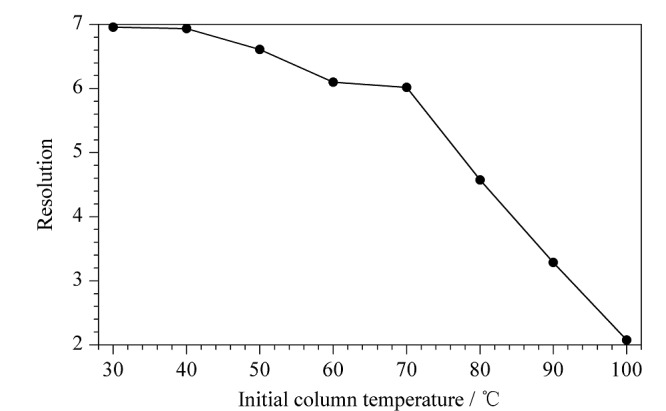
(*R*)-和(*S*)-4-氯甲基-2,2-二甲基-1,3-二氧戊环 在不同初始柱温下的分离度

### 2.4 升温速率对分离度的影响

本文考察了升温速率为1.0、1.5、2.0、2.5、3.0、3.5和4.0 ℃/min时4-氯甲基-2,2-二甲基-1,3-二氧戊环对映异构体的分离效果,见图5。结果表明,当升温速率高于2.0 ℃/min时,(*R*)-和(*S*)-4-氯甲基-2,2-二甲基-1,3-二氧戊环的分离度明显下降,当升温速率低于2.0 ℃/min时,两者间的分离度不再增加,但随着升温速率的降低,两个化合物的色谱峰会展宽,导致峰高降低,进而影响灵敏度。因此,最终选择升温速率为2.0 ℃/min。

**图5:**
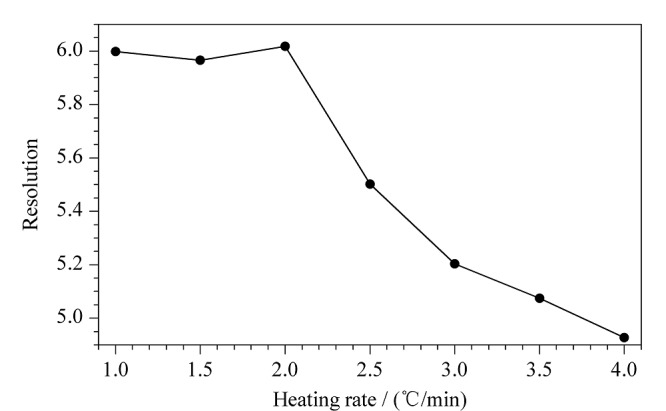
(*R*)-和(*S*)-4-氯甲基-2,2-二甲基-1,3-二氧戊环 在不同升温速率下的分离度

### 2.5 溶样溶剂对分离度的影响

本实验考察了溶样溶剂对(*R*)-和(*S*)-4-氯甲基-2,2-二甲基-1,3-二氧戊环分离度的影响。分别选用甲醇、乙醇、乙酸乙酯、正己烷、二氯甲烷、二甲基亚砜作为溶样溶剂,分别配制质量浓度为200 mg/L的外消旋体溶液。结果表明,除二甲基亚砜会干扰目标化合物出峰外,其余溶剂作为溶样溶剂时对(*R*)-和(*S*)-4-氯甲基-2,2-二甲基-1,3-二氧戊环的分离度无明显影响,本文最终选择甲醇作为溶样溶剂。在优化后的最佳色谱条件下,即线速度为70 cm/s、初始柱温为70 ℃、升温速率为2.0 ℃/min的条件下,4-氯甲基-2,2-二甲基-1,3-二氧戊环对映体的拆分色谱图见图6。

**图6:**
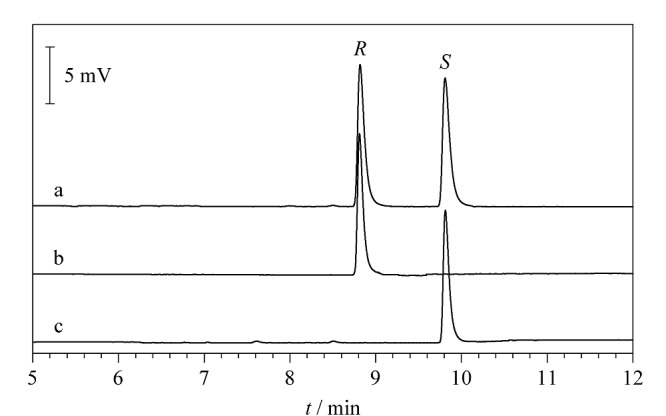
4-氯甲基-2,2-二甲基-1,3-二氧戊环 对映体的拆分色谱图

### 2.6 线性范围、检出限及定量限

取不同浓度的混合标准工作溶液进样,以质量浓度为横坐标(*X*, mg/L),对应的峰面积为纵坐标(*Y*)绘制校准曲线。所得校准曲线的线性范围、线性方程、相关系数(*r*^2^)见表1。检出限(LOD)和定量限(LOQ)分别以3倍*S/N*和10倍*S/N*确定。结果表明,(*R*)-和(*S*)-4-氯甲基-2,2-二甲基-1,3-二氧戊环在0.5~50.0 mg/L的范围内线性关系良好,相关系数大于0.998。(*R*)-和(*S*)-4-氯甲基-2,2-二甲基-1,3-二氧戊环的LOD分别为0.07 mg/L和0.08 mg/L,LOQ分别为0.22 mg/L和0.25 mg/L。

**表1:** (*R*)-和(*S*)-4-氯甲基-2,2-二甲基-1,3-二氧戊环的线性范围、线性方程、相关系数、检出限和定量限

Compound	Linear range/(mg/L)	Linear equation	*r*^2^	LOD/(mg/L)	LOQ/(mg/L)
(*R*)-	0.5-50.0	*Y*=10352.1*X*-1845.7	0.9983	0.07	0.22
(*S*)-	0.5-50.0	*Y*=10139.1*X*-1738.3	0.9987	0.08	0.25

*Y*: peak area; *X*: mass concentration, mg/L.

### 2.7 加标回收率和精密度

精密量取适量(*R*)-和(*S*)-4-氯甲基-2,2-二甲基-1,3-二氧戊环标准储备液,用甲醇配制成0.5、2.0、10.0 mg/L(以单独构型的浓度计)3个不同水平的混合样品溶液,上机依法测定,每个水平测定3次,计算加标回收率(见表2)。结果表明,(*R*)-和(*S*)-4-氯甲基-2,2-二甲基-1,3-二氧戊环的加标回收率分别为94.0%~99.1%和96.0%~98.8%,相对标准偏差(RSD)分别为1.26%~4.87%和1.51%~4.46%。

**表2:** (*R*)-和(*S*)-4-氯甲基-2,2-二甲基-1,3-二氧戊环 在3个水平下的加标回收率(*n*=3)

Compound	Background	Spiked/ (mg/L)	Found/ (mg/L)	Recovery/ %	RSD/ %
(*R*)-	ND	0.5	0.47	94.0	4.87
	ND	2.0	1.89	94.5	3.62
	ND	10.0	9.91	99.1	1.26
(*S*)-	ND	0.5	0.48	96.0	4.46
	ND	2.0	1.92	96.0	2.63
	ND	10.0	9.88	98.8	1.51

ND: not detected.

在最佳色谱条件下,对质量浓度为2.0 mg/L的混合标准品溶液连续进样6次,计算(*R*)-和(*S*)-4-氯甲基-2,2-二甲基-1,3-二氧戊环保留时间和峰面积的RSD。根据实验结果可知,两种化合物的保留时间RSD均为0.03%,峰面积RSD均小于2%,表明仪器精密度良好。

## 3 结论

对映异构体的分离基于“手性识别”原理,即固定相手性选择剂和对映异构体形成加合物,导致瞬态非对映异构体复合物的形成。但是瞬态非对映异构体复合物之间的差异依然非常微小,通过GC柱非常高的理论塔板数来放大差异,才能将对映异构体进行拆分。手性毛细管气相色谱法是一种非常简单、快速、方便、高效的分析方法,适用于拆分测定具有挥发性和热稳定性的对映异构体。本文采用手性毛细管气相色谱法,对手性医药中间体4-氯甲基-2,2-二甲基-1,3-二氧戊环对映异构体进行了拆分,考察了常规聚硅氧烷类、聚乙二醇类色谱柱及4款手性环糊精衍生物色谱柱对该对映异构体的拆分效果。此外,考察了线速度、初始柱温、升温速率、溶样溶剂等条件对该对映异构体分离度的影响。本方法不仅解决了4-氯甲基-2,2-二甲基-1,3-二氧戊环对映异构体的拆分难题,同时也为其他手性化合物的拆分提供了气相色谱的方法开发思路。然而,本文着重于气相色谱分离条件的优化,未对该对映异构体手性拆分的深层机理进行讨论。
